# Editorial: Environmental Enrichment as a Treatment? Epigenetic Mechanisms, Challenges and Limitations

**DOI:** 10.3389/fphar.2021.658970

**Published:** 2021-04-23

**Authors:** Rosa Redolat, Patricia Mesa-Gresa, Patricia Sampedro-Piquero, Debora Cutuli

**Affiliations:** ^1^ Departamento de Psicobiología, Facultad de Psicología, Universitat de València, Valencia, Spain; ^2^ Laboratorio de Neurociencias, Facultad de Psicología, Universidad de Oviedo, Oviedo, Spain; ^3^ Psychology Department, Sapienza University of, Rome, Italy

**Keywords:** environmental enrichment (EE), epigenetic, DNA, treatment strategies, translational, aging

Environmental enrichment (EE) is an improvement in the housing conditions of the laboratory animals by providing physical, cognitive, sensorial and social stimulation. Although different EE protocols exist, most of them consist of increasing the size of the cage, provide running wheels and novel and attractive objects to the animals (Robison et al., 2020). This type of protocol has shown important benefits, not only on the welfare, mood and cognition of healthy animals, but also on experimental models of different pathological conditions, such as neurodegenerative diseases, traumatic brain injury, epilepsy, cancer, stress and depression (Solinas et al., 2021). Nevertheless, the neurobiological mechanisms by which EE induces these benefits are yet highly unknown. Some recent experimental studies have provided information regarding changes in neural activity, whereas others have started to focus on epigenetic mechanisms involved in the brain changes related to exposure to EE (Tang, 2020). On the other hand, gene-environment interactions may be an intermediary step in providing an appropriate response to the environmental conditions leading to changes in behavior, emotion or cognition (Rogers et al., 2019). For instance, there is some evidence that environmental factors are able to promote changes in DNA methylation in a gene/promoter-specific manner and this process seems to play an important role in controlling gene expression involved in learning and memory or hypothalamic-pituitary axis functioning (See Figure 1).

**FIGURE 1:**
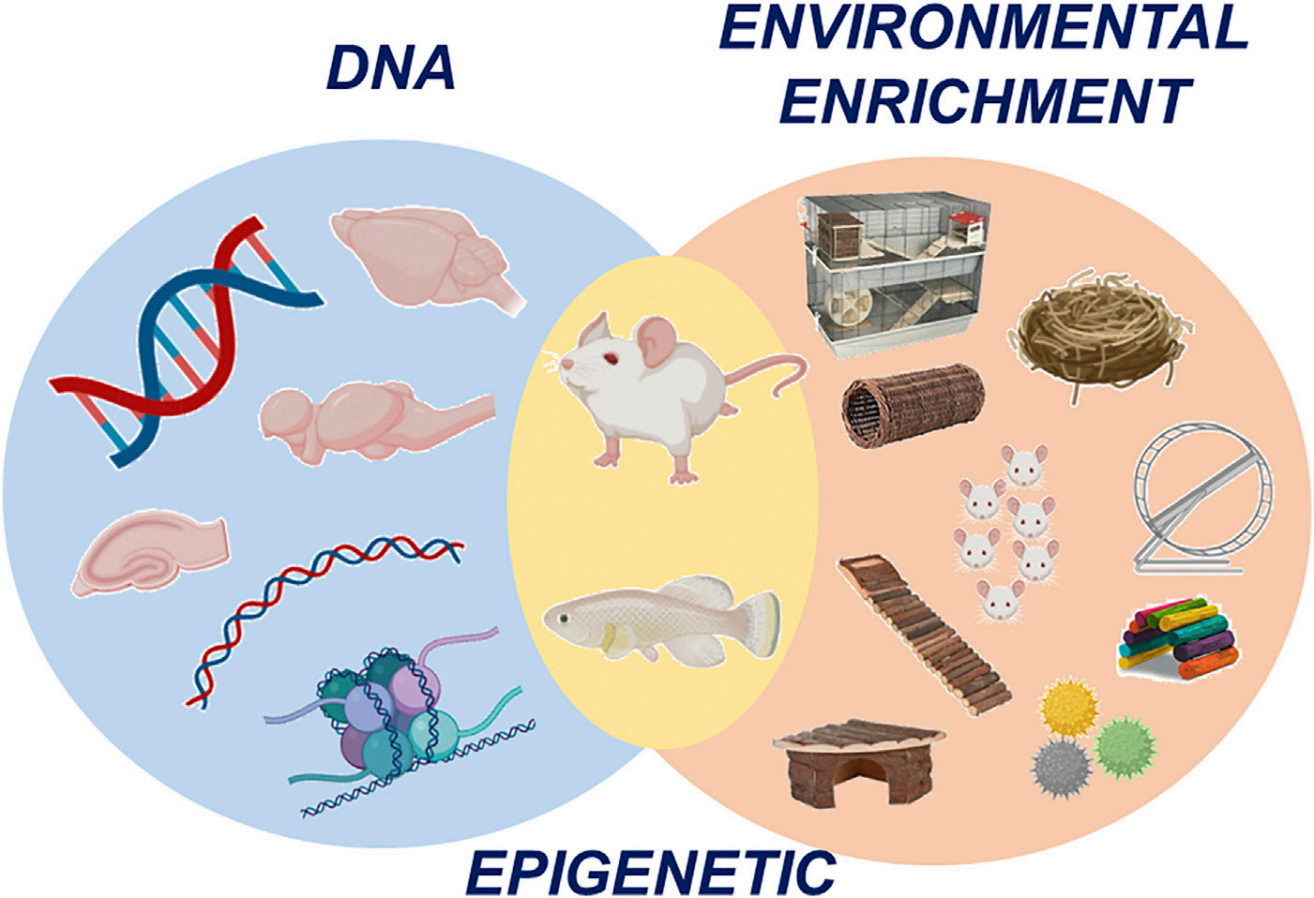
The diagram displays the interaction between environmental enrichment and epigenetic mechanisms. The present research topic addresses the question of how basic experimental research in different species can contribute to the development of therapeutic applications of enriched environments (Figure elaborated with BioRender).

The benefits of EE on learning and memory processes, as well as in the underlying brain plasticity, also occur in other species. Regarding this, the manuscript of Pereira et al. show that the visuospatial and somatomotor stimulation provided by EE in *Colossoma macropomum* fishes was able to improve spatial learning and memory rates, along with a greater number of cells in the telencephalon of adult fishes maintained in enriched conditions. Moreover, their study also evaluated differential gene expression using brain transcriptomics. It is a promising avenue, which revealed that EE increased the density of thrombocytes and lymphocytes. These findings could be a starting point for exploring potential genetic regulation mechanisms underlying better learning and memory performance, increased proliferative activity of the telencephalon, and peripheral blood differences.

Different factors, as the strain of the animals, their genetic background, sex, or period at which animals receive EE can influence on the results obtained both when EE is implemented alone or combined with other interventions (Mesa-Gresa et al., 2021). Studies presented in this RT reflect this diversity in the protocols applied to enriched environments. Hence, Rojas-Carvajal et al. present an interesting approach on EE effects depending on different experimental protocols. Specifically, authors consider that potential benefits of EE may vary according to the level of incentive salience. Results showed that restricted and unpredictable EE protocols led to a more rewarding effect in the animals compared to constant and prolonged enrichment conditions. This useful study reveals that motivation to explore the environment can be modulated based on EE characteristics. Moreover, the benefits observed after limited EE conditions on exploration-related behaviors, as well as on the sort of ultrasonic vocalizations in the open-field test seemed to be related to an enhancement of BDNF/TrkB expression.

Queen et al. reviewed cognitive and behavioral aspects of aging and suggest that EE-based animal models can help us to extend the health span and to improve cognitive status. Experimental studies can also contribute to obtain a better knowledge regarding epigenetic mechanisms and phenotypic changes involved in the aging process. Recent literature emphasizes the role of upregulation of the hypothalamic brain-derived neurotrophic factor (BDNF) after exposure to enriched environments. Taking into account results obtained in its own laboratory and others, Queen et al. shed light on how we can prevent or delay pathological age-related negative changes by means of environmental and lifestyle-based interventions. The review provides examples of the therapeutic value of enriched environments suggesting that factors such as the promotion of cognitive reserve, physical activity or social connection may have a significant clinical impact on successful aging. Results as those presented in this RT can also open new avenues for boosting the translation of results obtained from animal models of EE to clinical applications.

Manuscripts included in this editorial highlight two aspects of current research on the EE. On the one hand, the need to standardize the protocols due to the great variability between the different studies, in terms of duration, type of stimuli or frequency of exposure to novelty. Moreover, the characteristics of the animals themselves (species, age, pathological or health condition) are also a factor to be considered when interpreting results of experimental studies (MacArthur Clark, 2018). On the other hand, studies about the interaction between environmental conditions and the administration of different drugs with the aim of assessing epigenetic aspects is another question that recent work has addressed (Hannan, 2020). In general, we can conclude that recent research has suggested that EE can act as a therapy in different contexts and diseases, and these are interesting fields to be further explored in future studies. Furthermore, in the midst of Covid-19 pandemic, a better knowledge about how non-pharmacological interventions and lifestyle modifications can promote health and resilience, prevent neuroinflammation or improve immune function in the population is a topic that deserves attention (Muscat and Barrientos, 2020). In line with this, Brenes et al. have shown the potential of EE as a non-pharmacological treatment for depression. In this study, a differential antidepressant-like effect of exposure to various types of environment was observed. Specifically, adolescent rats were exposed to social isolation inducing a depressive-like phenotype. Then, the effectiveness of exposure to EE or physical activity was compared to the effect of fluoxetine treatment. The greatest antidepressant effect, assessed at behavioral and neurobiological levels, was found after exposure to EE, suggesting its beneficial effects.

Future research should also focus on individually tailored stimulation protocols to improve their effect and afford a better understanding of the underlying processes (Balietti et al., 2021). Finally, it is possible that restricted access to EE mimics many aspects of modern life in humans, such isolation of elderly population or the busy and stressful life we have, so that its potential positive effect as a model for non-pharmacological intervention can be highlighted.

## References

[B1] Balietti M. Pugliese A. Conti F. (2021). In aged rats, differences in spatial learning and memory influence the response to late-life Environmental Enrichment. Exp. Gerontol. 146, 111225. 10.1016/j.exger.2020.111225 33388381

[B2] Hannan A. J. (2020). Epimimetics: novel therapeutics targeting epigenetic mediators and modulators. Trends Pharmacol. Sci. 41 (4), 232–235. 10.1016/j.tips.2020.01.005 32008853

[B3] MacArthur Clark J. (2018). The 3Rs in research: a contemporary approach to replacement, reduction and refinement. Br. J. Nutr. 120 (s1), S1–S7. 10.1017/S0007114517002227 29081302

[B4] Mesa-Gresa P. Ramos-Campos M. Redolat R. (2021). Behavioral impact of experience based on environmental enrichment: influence of age and duration of exposure in male NMRI mice. Dev. Psychobiol. 10.1002/dev.22093 33452673

[B5] Muscat S. M. Barrientos R. M. (2020). Lifestyle modifications with anti-neuroinflammatory benefits in the aging population. Exp. Gerontol. 142, 111144. 10.1016/j.exger.2020.111144 33152515 PMC7704639

[B6] Robison L. S. Francis N. Popescu D. L. Anderson M. E. Hatfield J. Xu F. (2020). Environmental enrichment: disentangling the influence of novelty, social, and physical activity on cerebral amyloid angiopathy in a transgenic mouse model. Ijms 21 (3), 843. 10.3390/ijms21030843 PMC7038188 32012921

[B7] Rogers J. Renoir T. Hannan A. J. (2019). Gene-environment interactions informing therapeutic approaches to cognitive and affective disorders. Neuropharmacology 145 (Pt A), 37–48. 10.1016/j.neuropharm.2017.12.038 29277490

[B8] Solinas M. Chauvet C. Lafay-Chebassier C. Jaafari N. Thiriet N. (2021). Environmental enrichment-inspired pharmacological tools for the treatment of addiction. Curr. Opin. Pharmacol. 56, 22–28. 10.1016/j.coph.2020.09.001 32966941

[B9] Tang B. (2020). Axon regeneration induced by environmental enrichment- epigenetic mechanisms. Neural Regen. Res. 15 (1), 10–15. 10.4103/1673-5374.264440 31535635 PMC6862393

